# The Inversion of the Control Region in Three Mitogenomes Provides Further Evidence for an Asymmetric Model of Vertebrate mtDNA Replication

**DOI:** 10.1371/journal.pone.0106654

**Published:** 2014-09-30

**Authors:** Miguel M. Fonseca, D. James Harris, David Posada

**Affiliations:** 1 Department of Biochemistry, Genetics and Immunology, University of Vigo, Vigo, Spain; 2 CIBIO/InBIO, Research Center in Biodiversity and Genetic Resources, University of Porto, Vairão, Portugal; Ben-Gurion University of the Negev, Israel

## Abstract

Mitochondrial genomes are known to have a strong strand-specific compositional bias that is more pronounced at fourfold redundant sites of mtDNA protein-coding genes. This observation suggests that strand asymmetries, to a large extent, are caused by mutational asymmetric mechanisms. In vertebrate mitogenomes, replication and not transcription seems to play a major role in shaping compositional bias. Hence, one can better understand how mtDNA is replicated – a debated issue – through a detailed picture of mitochondrial genome evolution. Here, we analyzed the compositional bias (AT and GC skews) in protein-coding genes of almost 2,500 complete vertebrate mitogenomes. We were able to identify three fish mitogenomes with inverted AT/GC skew coupled with an inversion of the Control Region. These findings suggest that the vertebrate mitochondrial replication mechanism is asymmetric and may invert its polarity, with the leading-strand becoming the lagging-strand and vice-versa, without compromising mtDNA maintenance and expression. The inversion of the strand-specific compositional bias through the inversion of the Control Region is in agreement with the strand-displacement model but it is also compatible with the RITOLS model of mtDNA replication.

## Introduction

Mitochondrial DNA has been widely used as a molecular marker because of its maternal transmission, haploidy, limited recombination, high mutation rate and availability in animal cells. Additionally, the discovery that mutations in mtDNA can cause human diseases has increased the interest of the scientific community in understanding mtDNA evolution or its maintenance [1]. The latter consists of the processes that keep mtDNA viable, which in turn will have consequences at the cell biological and organismal level and is, in part, dependent of molecular processes such as mtDNA replication and transcription. Understanding these processes will undoubtedly improve our knowledge about mtDNA evolution itself.

The vertebrate mitogenome is typically a circular, double-stranded DNA molecule of ∼17 kb that encodes 13 proteins essential for the function of the respiratory chain as they constitute key components of the electron transport chain complexes required for oxidative phosphorylation. It also contains 2 ribosomal RNAs (12S and 16S rRNAs) and 22 transfer RNAs (tRNAs), which are associated with translation. In addition, mitochondrial genomes are known to have a strong strand-specific compositional bias [2] with the individual mtDNA strands being distinguished by its uneven guanine content: the heavy-strand (H-strand) is guanine rich whereas the light-strand (L- strand) is guanine poor [3].

When mutation and selection affect equally both DNA strands, nucleotide frequencies within each strand should be at equilibrium: A = T and G = C (Parity Rule type 2, PR2, [4]). Strand bias can be detected as deviations from this relationship, implying the existence of asymmetric mutational patterns that may result from different mutation rates, selective pressures or both, between the two strands of DNA [5]. However, the observation that the strand-specific bias is more pronounced at fourfold redundant sites of mtDNA protein coding genes (4-fold sites), where selective pressures are generally weaker than at other codon positions, suggests that strand asymmetries, to a large extent, are caused by mutational asymmetric mechanisms (e.g. DNA transcription, repair or replication). Two different hypotheses associate transcription with compositional bias: a strand-specific transcription-coupled repair (TCR) mechanism or strand-specific transcription rates. However, TCR *per se* in vertebrate mitochondria has not been described [6], [7]. Transcription is a strand-specific process (transcribed vs. non-transcribed strand) and thus might be a possible cause for the observed differences between strands. During transcription, the non-transcribed strand becomes transiently single-stranded and exposed to DNA damage while repair enzymes act on the transcribed strand [8], [9]. Since both mtDNA strands are transcribed as single/large polycistronic units, one would expect a constant mutation rate and a similar compositional bias along the genome. However, this is not compatible with the gradient of the mutation rate and of the compositional bias observed along mitochondrial genomes [10]–[14]. Altogether, replication and not transcription seems to play a major role in shaping compositional bias in vertebrate mitogenomes. Transcription may still influence the nucleotide composition variation of both strands, but to a lesser extent.

**Figure 1:**
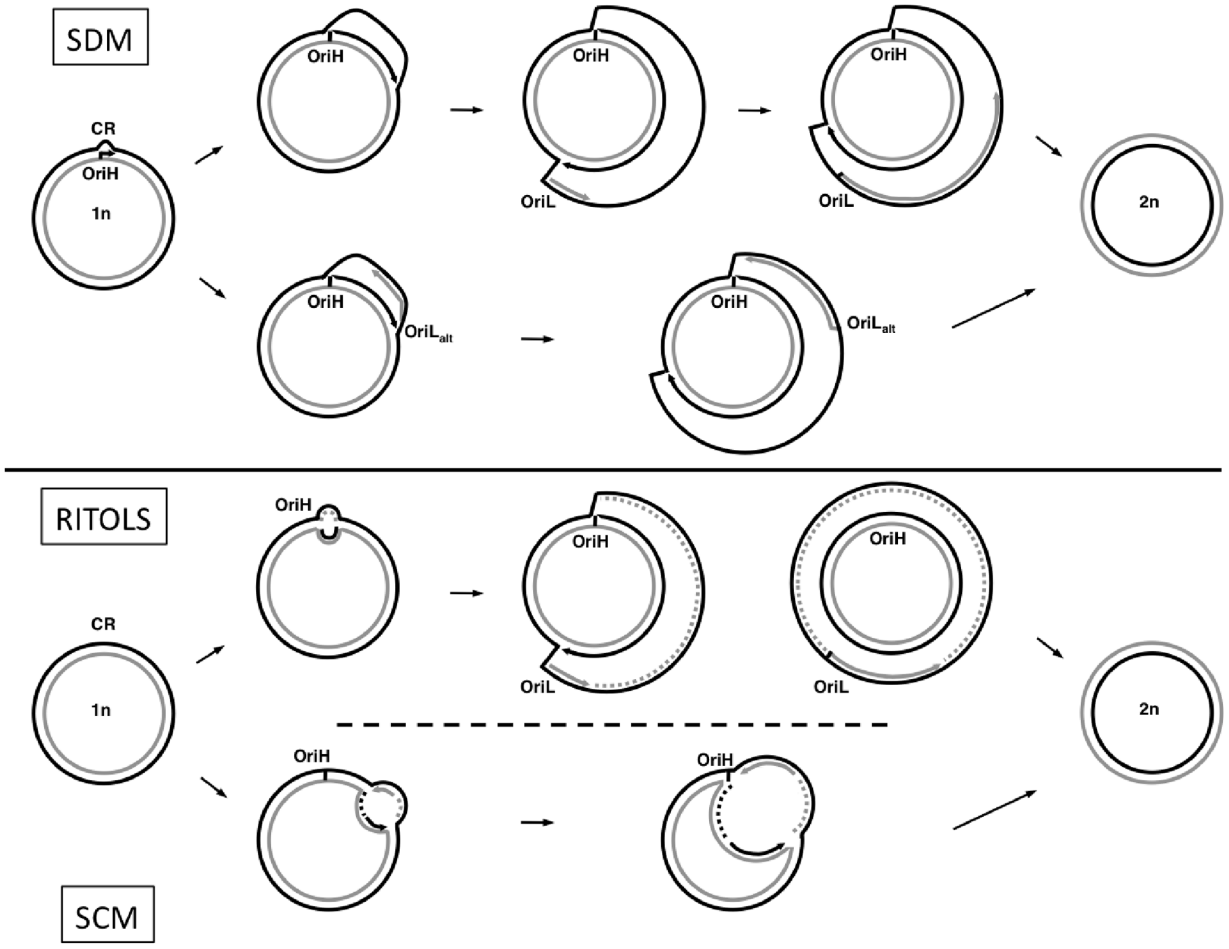
Models of mtDNA replication in vertebrates. Heavy-strand and Light-strand represented in black and grey, respectively. **(SDM)** In the strand-displacement model, replication of the H-strand initiates at OriH and is synthesized unidirectionally (black arrow), displacing the parental H-strand as single-stranded DNA. When OriL (or OriLalt) is exposed, the L-strand synthesis initiates in the opposite direction (grey arrow). **(RITOLS)** In the Ribonucleotide incorporation throughout the lagging strand model, synthesis of the H-strand initiates at a discrete origin (OriH) and proceeds unidirectionally (black arrow), displacing the parental H-strand. The L-strand is initially laid down as RNA (grey dashed line) using the displaced H-strand as template. The RNA is subsequently replaced with DNA (gray arrow). The maturation step usually starts at the OriL. **(SCM)** In the strand-coupled model, initiation of both H-strand and L-strand synthesis occurs bidirectionally from multiple origins across a broad zone downstream of OriH. This is followed by progression of both forks until the forks arrest at OriH. Black or grey dashed lines represent newly synthesized DNA Okazaki fragments.

For decades, mitochondrial DNA (mtDNA) replication was thought to occur through strand-displacement (the strand-displacement model, SDM; Figure 1 [15]–[18]), where both strands, the H-strand and the L-strand are replicated continuously, asymmetrically, unidirectionally and asynchronously from two different origins of replication - one for each strand - OriH (H-strand replication origin, located in the main mtDNA non-coding fragment, named the Control Region, CR) and OriL (L-strand replication origin). The synthesis of the nascent H-strand initiates at the OriH, and when its replication fork reaches the region where the OriL is located (about 2/3 of the molecule away from OriH), the OriL becomes single-stranded and forms a stem-loop structure that promotes the replication of the L-strand in the opposite direction [19]–[22]. According to the SDM, while the daughter H-strand is being synthesized, the parental H-strand becomes single-stranded and therefore more exposed to mutagenic reactions than the L-strand [16].

At the beginning of this century, the proposal of an alternative model for mtDNA replication [23] (Figure 1), in which mtDNA synthesis occurs through a bidirectional coupled leading- and lagging-strand synthesis (strand-coupled model, SCM), triggered an active debate. Since then, several refinements of the SCM were proposed [24]–[27]. More recently, Yasukawa et al. [28] proposed a new model, named RITOLS (Ribonucleotide Incorporation ThroughOut the Lagging Strand), in which replication initiates unidirectionally from the CR. The H-strand is synthesized continuously, while the L-strand is replicated, through a maturation step from RNA to DNA, using provisional RNA segments that hybridized to the template H-strand. The maturation step is initiated at one or more preferred sites, with the OriL region being a prominent site at maturation initiation. The RITOLS model has several features in common with the SDM. Both models propose that DNA replication is unidirectional, continuous and initiated at OriH, both predict a delay between the two DNA strands synthesis and both consider that the OriL is a major initiation site of the lagging-strand synthesis. The main difference between RITOLS and SDM models is that, during the prolonged delay between initiation of leading- and lagging-strand DNA synthesis, the parental leading-strand (or H-strand) is hybridized to RNA in the RITOLS model, whereas in SDM it remains single-stranded. Although recent biochemical studies support both the RITOLS model [29], [30] and SDM [20], the RITOLS model has gained additional support in later years. Several biochemical studies, using electron microscopy techniques, immunopurification with antibodies specific to RNA:DNA hybrids [29] or *in organello* DNA synthesis [30], showed that extensive RNA:DNA duplexes are present *in vivo* [30] and mainly found in one strand during replication [29]. Also, the SCM replication intermediates with duplex DNA seem to be better understood as the result of the maturation of RNA:DNA duplexes into DNA:DNA duplexes of the RITOLS model [31].

Although at the biochemical level the model for vertebrate mtDNA replication is still a debated issue, from a molecular evolutionary perspective, three features of vertebrate mtDNA are commonly associated with an asymmetric mechanism, with two distinct replication origins:

### i) Compositional/mutational bias gradient along the genome

Many studies have shown or suggested that specific mutations occur preferentially on the H-strand [10]–[13], [32], [33]. However, this exposure to mutagenesis is not uniform along the H-strand with some regions of the genome being more susceptible to damage than others. This exposure gradient leads to a gradient in nucleotide substitution, which ultimately results in a gradient of asymmetric base composition in both strands [11], [12]. This can be explained as a consequence of a strand asymmetric mode of replication. For example, according to SDM model of mtDNA replication, the lagging-strand synthesis should initiate at OriL and then the replication fork should replicate unidirectionally and continuously the nascent lagging-strand until it reaches again the OriL region. The regions closest to OriL in the direction of the lagging-strand replication fork will be exposed in ssDNA only for a short period of time because these will be the first regions of the daughter lagging-strand to be synthesized. On the contrary, the regions adjacent to the OriL, but farthest in the direction of the lagging-strand replication fork, will be exposed in ssDNA for the longest period during replication because they will be the last regions of the daughter lagging-strand to be synthesized [11], [13]. Thus, there is a gradient along the genome in the duration of time spent as ssDNA [10], [11], [32] with only two abrupt changes around both origins of replication. The hypothesis of the correlation between the time spent in ssDNA and the asymmetry in base composition was derived from mutational studies on the relative rates of single-strand and double strand mutations [34]–[37]: in ssDNA there is an increased rate of hydrolytic deamination of cytosine (C→T) and of adenine (A→G) that leads to an accumulation of thymine and guanine on the exposed strand. This is consistent with the observed compositional bias found in vertebrate mtDNA with the H- or leading-strand being TG-rich. The spectrum and strand-specific asymmetry of the mtDNA mutation accumulation inferred in population studies were recently supported at the somatic level [33]. However, the argument that, in SDM, the regions furthest from OriL and closest to OriH will have more mutations because they spend more time as ssDNA might apply equally to RITOLS, except that it spends the time as RNA/DNA hybrid.

### ii) OriL sequence and secondary structure conservation across most vertebrates

The vertebrate mtDNA is a very compact genome and usually nonfunctional sequences are rapidly eliminated [38], while functionally active structures are conserved across divergent taxa. The OriL falls into the latter category because, although very small, it has conserved sequence elements and maintains its ability to form a hairpin-structure throughout most vertebrates [20]. The fact that some vertebrate groups lack a recognizable OriL in its typical location [20] suggests that the mtDNA replication in these species might use the same replication origin for both strands. Another possibility, as suggested by biochemical [18] and computational studies [39] is that replication could use alternative OriLs. Comparative analyses have showed that compositional bias changes abruptly around the OriL position, which led some to suggest that this structure may function as replication origin in a SDM-like mechanism for mtDNA replication [13], [14], [40]. The importance of OriL in mitochondrion functionality has been reinforced by a recent *in vivo* mutational saturation study of mtDNA in mutator mice in which mutations in OriL were selected against [20] (mutator mice accumulate higher levels of mtDNA somatic mutations because they carry a proofreading-deficient form of the mtDNA polymerase γ that is error-prone in the course of replication). Again, the argument that the OriL is a key structure for SDM applies equally to the RITOLS model.

### iii) Inversion of the strand-specific compositional bias through CR inversion

It was suggested in previous studies, in invertebrates [41]–[43] and in one vertebrate species [44], that the inversion of the coding polarity of the sequence elements that regulate and initiate replication caused the inversion of the strand-compositional bias. According to the authors, with the inversion of the CR the leading-strand (H-strand) becomes the lagging-strand and vice-versa. Assuming the SDM as the main replication process, the L-strand in these mitogenomes is the strand being exposed to mutational damage and not the H-strand. With the replication inversion and given enough evolutionary time, the strand-specific compositional bias also inverts accordingly to the new state each strand has during replication (*exposed vs. non-exposed strand*). This scenario might apply also to the RITOLS model if the mutational spectrum expected for RNA:DNA duplexes is similar to the one observed for ssDNA.

In this study, we analyzed almost 2,500 complete vertebrate mitogenomes in order to search for new and independent CR inversions and/or to detect new strand-specific compositional inversions. We found two novel and independent CR inversions in three fish species. The mitogenomes having this rare genome rearrangement also have inverted the strand-specific compositional bias. These results are consistent with *in vitro/vivo* findings [20], [29], [30], [45] and previous evolutionary genetic studies [11], [13], [33] that suggest that vertebrate mtDNA replicates either following the SDM or the RITOLS models.

## Results

### Vertebrate mtDNA strand-specific compositional bias

**Figure 2:**
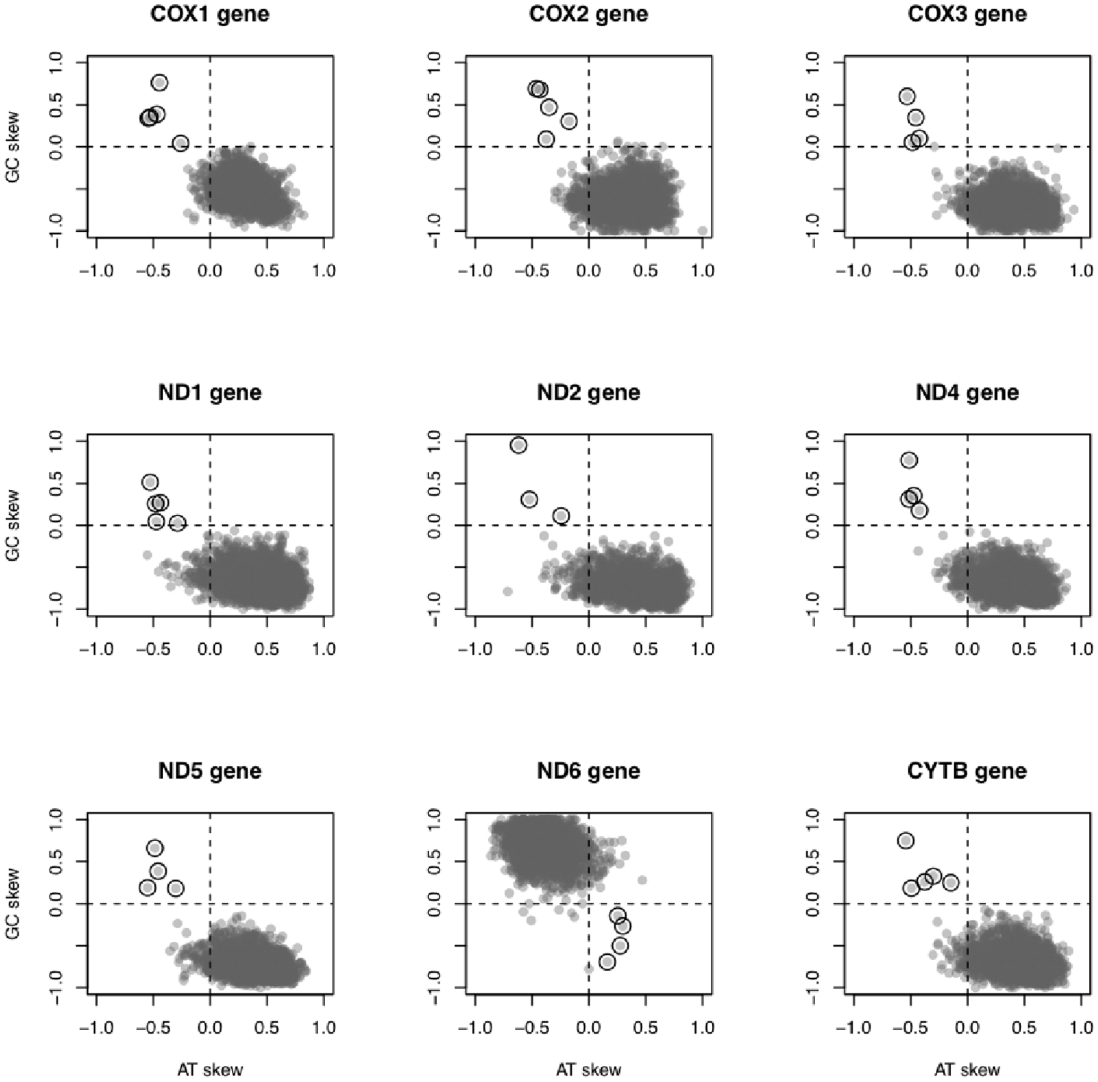
Plot of species-specific GC and AT skew at 4-fold sites at mitochondrial protein-coding genes. ND3, ND4L, ATP8 and ATP6 were not included in the analysis (see Material and Methods). Empty circles highlight genes with inverted AT and GC skews, all of them belonging to one of the following mitogenomes: *Albula glossodonta* (Albuliformes: Albulidae, NCBI code NC_005800, AT and GC skews inversion in 9 of 9 genes), *Bathygadus antrodes* (Gadiformes: Macrouridae, NCBI code NC_008222, AT and GC skews inversion in 4 of 9 genes), *Tetrabrachium ocellatum* (Lophiiformes: Tetrabrachiidae, NCBI code NC_013879, AT and GC skews inversion in 9 of 9 genes), two *Johnius* species (Perciformes: Sciaenidae, *J. grypotus* and *J. belangerii*, NCBI codes NC_021130 and NC_022464, AT and GC skews inversion in 8 and 9 of 9 genes, respectively).

For genes encoded on the H-strand, A and C were the most abundant nucleotides at 4-fold sites (most frequent 4-fold site: 75.2% A, 23.0% C, 1.8% T and <0.1% G). For ND6, the only protein-coding gene encoded on the L-strand in vertebrate mitogenomes, T and G were most abundant at 4-fold sites (most frequent 4-fold site: 71.1% T, 27.9% G, 1.0% A and 0% C). Five fish mitogenomes showed inverted skews (i.e., AT skew>0 and GC skew <0 for ND6; AT skew <0 and GC skew>0 for all remaining genes; Figure 2) for most or all nine protein-coding genes analyzed: *Albula glossodonta* (Albuliformes: Albulidae, NCBI code NC_005800 [46], AT and GC skew inversion in 9 of 9 genes), *Bathygadus antrodes* (Gadiformes: Macrouridae, NCBI code NC_008222 [47], AT and GC skew inversion in 4 of 9 genes), *Tetrabrachium ocellatum* (Lophiiformes: Tetrabrachiidae, NCBI code NC_013879 [48], AT and GC skew in 9 of 9 genes), two *Johnius* species (Perciformes: Sciaenidae, *J. grypotus* and *J. belangerii*, NCBI codes NC_021130 [49] and NC_022464 [50], AT and GC skew in 8 and 9 of 9 genes, respectively). Additionally, we calculated the cumulative AT and GC skews along the genome sequences to test whether the compositional bias inversion could also be detected using the whole mtDNA sequence and not only the 4-fold sites. Cumulative AT and GC skews have typically positive and negative slopes along the sequences but the five fish mitogenomes showed an inverted pattern (Figure S1 and Figure S2, Supporting Information).

### Control Region Reversed Translocation in mitogenomes with inverted AT/GC skews

**Figure 3:**
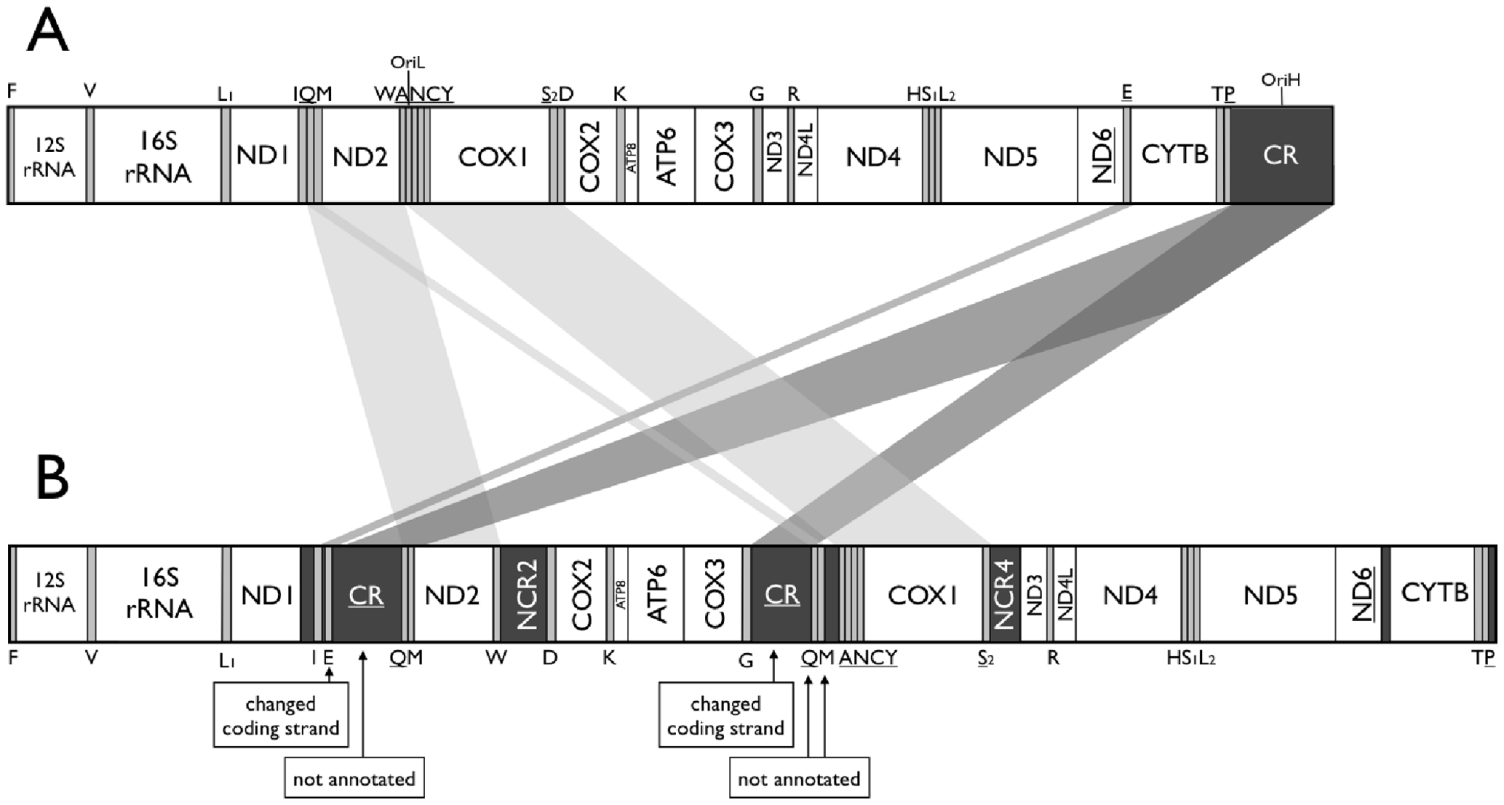
Linear representation of the organization of a mitogenome. (A) Typical gene organization of vertebrate mtDNA. (B) Gene organization of *Tetrabrachium ocellatum* mtDNA, in which the corrections to the original genbank file are highlighted. Light-strand encoded genes are underlined. Gene sizes are drawn relative to genome length. Gene abbreviations used are: ND1–6, NADH dehydrogenase subunits 1–6; COX1–3, cytochrome oxidase subunits I–III; ATP6 and ATP8, ATPase subunits 6 and 8; CYTB, cytochrome b; and one-letter codes of amino acids, tRNA genes specifying them (L1 and L2 for leucine tRNA genes specifying, respectively, UUR and CUN codons and S1 and S2 for serine tRNA genes specifying, respectively, AGY and UCN codons). CR, OriH, and OriL stand for the control region, the heavy-strand replication origin, and the light-strand replication origin, respectively. The bars connecting both mtDNA linear representations indicate relevant gene rearrangements (light grey) and inversions (dark grey).

Inverted skews were already known for *Albula glossodonta* and *Bathygadus antrodes* [50], so we conducted further analyses only with the mitogenomes of *T. ocellatum* and *Johnius* spp. A common feature of these three mitogenomes is that they lack the typical vertebrate mtDNA gene order [48]–[50]. They also have variable copy number of non-coding regions (NCR): 3 (*J. grypotus*), 4 (*T. ocellatum*), and 5 (*J. belangerii*). Of these NCR we identified 2 CR in the mitogenomes of *T. ocellatum* and of *J. grypotus* and 1 CR in the mitogenome of *J. belangerii.* Notably, the direction of these CRs was the opposite of all the CR of closely related species (Table 1). In *T. ocellatum* mitogenome tRNA-Glu inverted its transcription polarity (coding strand inversion determined by ARWEN and MiTFi (*E-value = 3.30e-10*), Figure 3), which is contrary of its original GenBank annotation.

**Table 1:** Statistical significance test of sequence similarity performed between Non-Coding Regions.

First query sequence^a^	Second query sequence	Direction	Smith-Waterman score	E-value^b^
*T. ocellatum* NCR1	*T. ocellatum* NCR3	forward	4080	5.60E-199
*T. ocellatum* NCR 1^c^	*Chaunax pictus*	**reverse**	758	1.80E-33
	*Ceratias uranoscopus* e	**reverse**	661	1.50E-34
	*Coelophrys brevicaudata*	**reverse**	590	3.50E-25
*Chaunax pictus*	*Ceratias uranoscopus* e	forward	1707	1.90E-100
	*Coelophrys brevicaudata*	forward	1039	2.10E-41
*Ceratias uranoscopus* e	*Coelophrys brevicaudata*	forward	910	1.00E-41
*J. grypotus* NCR1^d^	*J. grypotus* NCR3	forward	3232	1.00E-85
	*Argyrosomus japonicus*	**reverse**	443	2.00E-17
	*Bahaba taipingensis*	**reverse**	485	1.50E-19
	*Miichthys miiuy*	**reverse**	443	1.30E-16
*J. belangerii* NCR3	*Argyrosomus japonicus*	**reverse**	469	1.80E-19
	*Bahaba taipingensis*	**reverse**	548	8.70E-24
	*Miichthys miiuy*	**reverse**	528	6.40E-21
*J. grypotus* NCR1	*J. belangerii* NCR3	forward	1371	1.10E-45
*J. grypotus* NCR3	*J. belangerii* NCR3	forward	1450	2.50E-25
*Argyrosomus japonicus*	*Bahaba_taipingensis*	forward	2830	8.40E-145
	*Miichthys_miiuy*	forward	2807	1.20E-135
*Bahaba taipingensis*	*Miichthys_miiuy*	forward	3368	9.10E-166
*J. grypotus* NCR1^d^	*T. ocellatum* NCR1^c^	forward	286	2.10E-08
*J. belangerii* NCR3	*T. ocellatum* NCR1^c^	forward	291	1.90E-08
*Chaunax pictus*	*Argyrosomus japonicus*	forward	1722	4.60E-80
	*Bahaba taipingensis*	forward	1831	3.40E-85
	*Miichthys miiuy*	forward	1895	1.50E-84
*Coelophrys brevicaudata*	*Argyrosomus japonicus*	forward	1056	4.00E-48
	*Bahaba taipingensis*	forward	1068	1.10E-49
	*Miichthys miiuy*	forward	1052	4.30E-48
*Ceratias uranoscopus* e	*Argyrosomus japonicus*	forward	1283	1.40E-66
	*Bahaba taipingensis*	forward	1321	5.10E-69
	*Miichthys miiuy*	forward	1303	5.90E-67

a Tests of sequence similarity were performed between the NCRs of the three exceptional fish mitogenomes and the NCR of other closely related fish species.

b Only results with E-value<1.0E-05 are shown.

c *T. ocellatum* NCR3 showed similar results.

d *J. grypotus* NCR3 showed similar results.

e Mitogenome with two nearly identical CRs. Only one was included in this analysis.

### OriL identification in mitogenomes with inverted AT/GC skews

**Figure 4:**
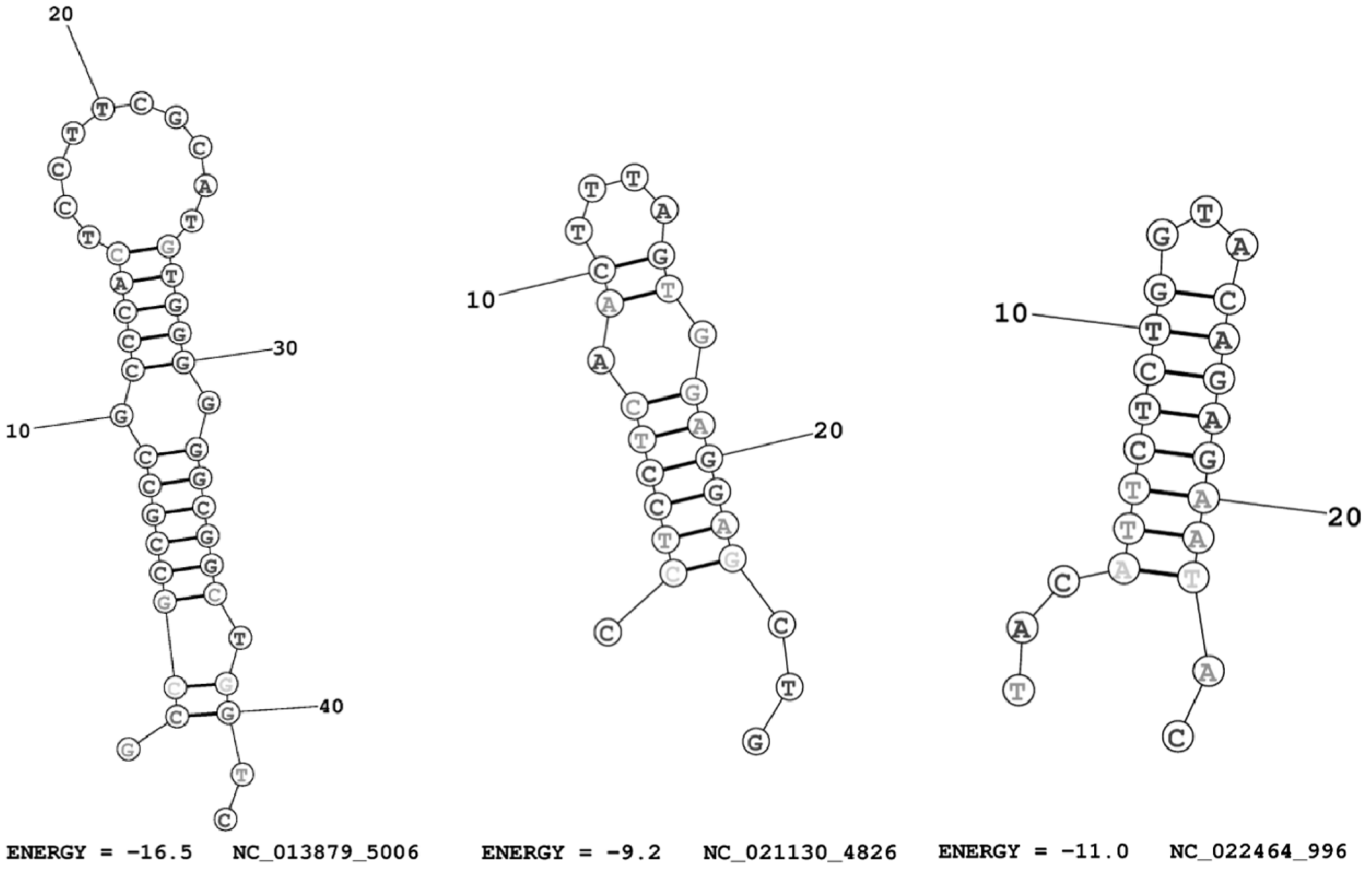
Examples of OriL-like structures identified in the three mitogenomes with CR inversion. The predicted OriL were searched in the L-strand because we assume that replication is inverted in these mitogenomes. The NCBI code of the mitogenome where the structure was predicted, the starting position and the thermodynamic entropy of each structure is specified below the figures. Left figure, *Tetrabrachium ocellatum* (Lophiiformes: Tetrabrachiidae, NCBI code NC_013879); Center figure, *Johnius grypotus* (Perciformes: Sciaenidae, NC_021130); Right figure, *Johnius belangerii*, NC_022464 (Perciformes: Sciaenidae, NC_021130). Pictures were drawn using RNAstructure Web Servers (http://rna.urmc.rochester.edu/RNAstructureWeb/).

In the three mitogenomes of the species with inverted CR the OriL sequence is shorter (from 7 to 26 nucleotides, instead of around 33-36) and it is not able to form a stable hairpin structure. The only stable hairpin at the OriL region that we found was in the mitogenome of *J. grypotus*, but it showed very low thermodynamic entropy for a typical OriL (−2.2 kcal/mol instead of around −11.0 kcal/mol), which suggests that this OriL is probably non-functional. We found several stable OriL-like structures for each mitogenome, although we could not determine with confidence the most likely putative OriL given the sequence variability they presented (Figure 4). The two mitogenomes with inverted CR identified in a previews study [44] also had no identifiable OriL (*A. glossodonta*, very short sequence of 6 nucleotides) or it was less stable than a typical OriL (*B. antrodes*, thermodynamic entropy = −7.10 kcal/mol).

## Discussion

In this study we describe three mtDNA CR inversions found in three species. Two of these species, are likely to share the same ancestral CR inversion because they are phylogenetically very closely related (belonging to the same genus) and also because these inversions are extremely rare. In one of the mitogenomes with novel CR inversions found here, the tRNA-Glu is translocated and also inverted from its original position. Most gene rearrangements in vertebrate mitogenomes have been ascribed to the tandem duplication/random loss model [51], [52]. This model can explain the extra CR copies or rearranged tRNAs in a given genome, but it does not account for arrangements involving a change in gene orientation, as it is the case here. One mechanism that could explain gene inversions is the head-to-head dimerisation of linearised monomeric mitogenomes [53]. According to this, the initial two copies of each gene would have opposite transcriptional polarities. Then, the inactivation of one of the two regions (CR) that regulate transcription would result in a gene arrangement in which all genes would have the same transcriptional direction. However, this mechanism cannot be applied to the gene inversions described here because the mitogenomes that suffered the CR inversions do not have all genes coded in the same strand, as expected by the head-to-head dimerisation of linearised monomeric model. In our opinion, homologous (or intra-mitochondrial) DNA recombination is the most plausible explanation for the gene inversions found here for the CR, because such mechanism allows for the inversion of partial genome fragments [54]. As far as we know, only three events of gene or fragment inversions have been reported in vertebrate mitogenomes so far. Amer and Kumazawa [55] observed a tRNA-Pro gene inversion in the lizard *Calotes versicolor*. Kong et al. [56] found an inversion of the tRNA-Gln in the mitogenome of the fish *Cynoglossus semilaevis*, which is common to several species of the Cynoglossidae family [57]. Finally, we previously reported the first CR inversion coupled with translocation [44]. In all three cases, homologous DNA recombination was identified as the putative mechanism originating the inversion.

Our results suggest that the correct identification of a non-coding region as the CR (or D-loop) should not be based solely on its size, AT content or presence of tandem repeats. CR identification should be supported by sequence similarity against known CRs of closely related species. We believe that the occurrence of regulatory motifs should be used to recognize a non-coding region as a putative Control Region. For this purpose, the Conserved Sequence Box II (CSB II), located in the 3′-end of the CR seems to be very appropriate because: i) CSB II plays a crucial role both in transcription and replication since it is required for the stability of the H-strand synthesis initiation [58], [59] and it acts as sequence-dependent termination element for transcription [59]; ii) it is conserved across different vertebrate groups [60], [61], which makes it easily identifiable within the AT-rich Control Region - CBS II is characterized by a poly-C stretch usually separated by a very small number (1-3) of T and A; and iii) probably its orientation can indicate which strand is the leading-strand during replication. The CR has also two more Conserved Sequence Blocks (CSB I and III), but their molecular role remains to be established [20] and they are less conserved and more difficult to identify [61]. In the fish mitogenomes with inverted CR identified here, the CSB II was present and unambiguously identified.

We here showed that it is possible to identify OriL-like structures across the mitogenomes, although it is then difficult to infer which one might be functionally active. OriL varies not only in its sequence composition but also in the size of its hairpin structure – number of basepairs in the stem or number of nucleotides in the loop [20]. The possibility that tRNAs genes can potentially function as alternative OriL adds some complexity to choose the most used one in the absence of the OriL in its typical location [39], [62], [63]. Wanrooij and colleagues [20] have developed an interesting method to identify OriLs across vertebrate mitogenomes based on sequence prediction coupled with hairpin structures detection. However, a few species still lack a recognizable OriL. On one hand, assuming that the OriL is essential for SDM and RITOLS replication mechanisms, how do these genomes lacking OriL replicate? On the other hand, if OriL plays no role in replication (SCM), why is this structure so remarkably conserved across most vertebrate groups? The observation that the mitogenomes with inversed CR lost a functional OriL in its typical location suggests that OriL may be indeed an active structure in replication [20]. If OriL were essential for mtDNA replication, then the CR inversion (and translocation) would also require the inversion and probably translocation of the OriL or the appearance of an alternative one to maintain an efficient replication process. In our opinion, more extensive comparative genomics analysis are needed to better understand the exact sequence requirements of a functional OriL - of the structure itself and of the immediate upstream and downstream regions.

The nucleotide frequencies that we measured for the 4-fold sites are consistent with the overall nucleotide compositional found both in H- and L- strands of vertebrate mitogenomes [2], [11], [13], [14]. These values also show that the majority of the protein-coding genes (96.3%) have the typical strand-specific compositional bias – positive AT skew and negative GC skew for those encoded on the H-strand and negative AT skew and positive GC skew for ND6. However, we found three new fish mitogenomes showing inverted AT/GC skew at 4-fold sites for most or all protein-coding genes (in addition to the two mitogenomes previously described [44]). The AT/GC skew inversion of a specific gene is common to occur in invertebrates if that gene changes its coding direction (i.e., a local inversion). Given enough evolutionary time, this inverted gene will change also the AT/GC skew in agreement with its new coding strand. However, if the AT/GC inversion involves most or even all protein-coding genes, an inversion of the mechanism that creates the strand-compositional bias seems more likely [41], [44].

Considering replication as the major source of AT/GC skew variation, which of the current mtDNA replication models could explain, from a molecular evolutionary perspective, the observed strand-specific skew and its inversion? Genomes with bidirectional replication (SCM-like) should have a V-shape distribution (inverted or not) of its cumulative skew diagrams [13]. However, cumulative skew diagrams of vertebrate mtDNA clearly do not follow this distribution. Abrupt changes in these diagrams (e.g. the switch in polarity in V-shape) can indicate the location of replication origins and terminus [13]. Indeed, vertebrate mtDNA show significant changes (but not a switch in polarity) near OriH [14] and OriL [13], [14], which suggests that both origins of replication play an active role during the process. However, the existence of OriL is not assumed in SCM. Altogether, these observations suggest that SCM replication does not occur in vertebrate mitogenomes.

One hypothesis to explain the global AT/GC skew gradient and its inversion is that mtDNA replicates through SDM. All the three fish mitogenomes described here present an inversion of the CR (and hence of the replication-related features), which is an indication of the inversion of the replication mechanism itself. Assuming a SDM mechanism, if the leading and exposed-strand changes, then the strand-specific mutational patterns will also change. Ultimately, the strand-specific compositional bias will also invert: genes that originally had positive AT skew and negative GC skew will invert those same biases, and vice-versa [44]. However, the same arguments to explain the global AT/GC skew inversion might also apply for the RITOLS model. This model is also asymmetric with the leading-strand being hybridized to RNA instead of being single-stranded during the process. An inversion of the RITOLS replication mechanism could also lead to an inversion of the strand-specific compositional bias if the mutational impact of the RNA:DNA duplexes were similar to the one that occurs for ssDNA and if the stability of RNA:DNA hybrids decreased with age.

Molecular evolutionary studies have traditionally favored the SDM over the RITOLS model because it is straightforward to link the SDM prediction for ssDNA with its high susceptibility to damage and its evolutionary impact on mtDNA: it is well-known that vertebrate mtDNA shows a strand-specific mutational bias that results in the accumulation of T and G on the leading-strand, and that this bias follows a gradient starting at the origin of replication [2], [10]–[12], [33], [44], [45], [64] (this study). The SDM easily explains this bias as a result of the time spent by the leading strand in ssDNA while is being synthesized, as different biochemical experiments have shown that ssDNA tends to accumulate T and G mutations [34]–[36], [65]. In addition, the spatial gradient of this bias is also easily explained by the SDM, in which regions located away from the origin of replication spend more time as ssDNA than more proximal regions. Because a smaller exposure as ssDNA is expected to reduce the mutational damage [66], the SDM predicts a positive correlation between the intensity of the bias and the distance to the OriL, which is what we see in real data [10], [11]. However, mitochondrial DNA replication is a very slow process that takes 1–2 hours to be completed [67], implying that the H-strand is exposed in ssDNA for a long period of time during each replicative cycle. Given the susceptibility of ssDNA to DNA damage, it is reasonable to expect that the cell would try to avoid such exposure, especially for long periods and in an environment with high concentration of reactive oxygen species. A RITOLS-like mechanism, with the formation of RNA/DNA hybrids, could have arisen in order to reduce the amount of DNA damage accumulated in the H-strand during replication, because these RNA/DNA duplexes are less susceptible to damage than ssDNA. However, from a molecular evolutionary perspective, it does not seem straightforward to link the observed mitogenome mutational/compositional bias and its inversion with a perfect RITOLS model, as we currently lack specific biochemical evidences suggesting that the duration of the RNA/DNA (leading-strand) hybrid is related to the amount of mutation or that these RNA/DNA duplexes are susceptible to the same mutational patterns as ssDNA is. Nevertheless, we should consider the possibility of occasional RITOLS errors in the formation RNA/DNA hybrids resulting in single-stranded regions in the lagging-strand, which might then explain the observed compositional bias [14], [68].

Future studies should aim to estimate both the spectrum and strand orientation asymmetry of mtDNA mutations related with the RITOLS mechanism to test whether they are consistent with the ones observed in somatic and germinal mtDNA evolution.

## Materials and Methods

We downloaded all 2,456 complete vertebrate mtDNA genomes available in GenBank (http://www.ncbi.nlm.nih.gov) in November 2013. For each mitogenome we measured the strand-specific compositional bias in terms of GC skew = (G-C)/(G+C), and AT skew = (A-T)/(A+T) [69] at 4-fold redundant sites of protein-coding genes using custom Perl scripts (available upon request). We examined the redundant codons of the following amino acids: alanine (GCN), proline (CCN), serine (TCN), threonine (ACN), arginine (CGN), glycine (GGN), leucine (CTN), and valine (GTN). We excluded the smallest protein-coding genes (ATP8, ND3 and ND4L) as they have few 4-fold sites and hence show high variance for AT/GC skews. ATP6 was also excluded because it considerably overlaps with ATP8. Four-fold sites located in overlapping region ATP8/ATP6 have strong selective constrains because they code for two different proteins and should not be considered “true” 4-fold sites. For the mitogenomes with inverted GC and AT skew we searched for tRNAs and identified its coding direction using ARWEN v1.2 [70] and MiTFi [71]. We tested for statistical significance of sequence similarity between tRNAs and between Control Regions using PRSS test (1000 uniform shuffles; [72], [73]). For this test we used sequences of the closest taxa with complete mitogenome sequence available when necessary.

For each mitogenome, we manually identified the OriL when present at the expected genomic location (between tRNA-Asn and tRNA-Cys), otherwise we predicted it. For the latter we extracted all possible mtDNA fragments from both strands from 15 up to 40 bp. For each fragment, we calculated the minimum free energy of each secondary structure using RNAstructure v.5.6 [74] (specified with DNA parameters). Only hairpin structures with the following OriL-like characteristic features were kept: minimum free energy below −8.0 kcal/mol; TC-rich 5′-end stem, with at least one C; 3′-end stem with at least one C, loop containing one or more Ts; and preference for no unpaired nucleotides within the stem.

## Supporting Information

Figure S1 **Plot of cumulative AT skew along the L-strand of 2726 vertebrate mitochondrial genomes.** The values were calculated according to the formula described in Grigoriev 1998, with a window size of 500 nucleotides and a window step of 25 nucleotides. Each line corresponds to the cumulative skew of an individual mitochondrial genome. We took into account the circularity nature of the molecule in this analysis. The sequences with Control Region inversion coupled with relevant inverted compositional bias at 4-fold sites are indicated: *Albula glossodonta* (Albuliformes: Albulidae, NCBI code NC_005800), *Bathygadus antrodes* (Gadiformes: Macrouridae, NCBI code NC_008222), *Tetrabrachium ocellatum* (Lophiiformes: Tetrabrachiidae, NCBI code NC_013879), two *Johnius* species (Perciformes: Sciaenidae, *J. grypotus* and *J. belangerii*, NCBI codes NC_021130 and NC_022464, respectively).
(DOCX)

Figure S2 **Plot of cumulative GC skew along the L-strand of 2726 vertebrate mitochondrial genomes.** The values were calculated according to the formula described in Grigoriev 1998, with a window size of 500 nucleotides and a window step of 25 nucleotides. Each line corresponds to the cumulative skew of an individual mitochondrial genome. We took into account the circularity nature of the molecule in this analysis. The sequences with Control Region inversion coupled with relevant inverted compositional bias at 4-fold sites are indicated: *Albula glossodonta* (Albuliformes: Albulidae, NCBI code NC_005800), *Bathygadus antrodes* (Gadiformes: Macrouridae, NCBI code NC_008222), *Tetrabrachium ocellatum* (Lophiiformes: Tetrabrachiidae, NCBI code NC_013879), two *Johnius* species (Perciformes: Sciaenidae, *J. grypotus* and *J. belangerii*, NCBI codes NC_021130 and NC_022464, respectively).
(DOCX)
