# Supplementary figures and images for: The Inversion of the Control Region in Three Mitogenomes Provides Further Evidence for an Asymmetric Model of Vertebrate mtDNA Replication
Source: PLoS One. 2014 Sep 30;9(9):e106654. doi: 10.1371/journal.pone.0106654 (PMC4182315; doi:10.1371/journal.pone.0106654)

**Figure S1.**


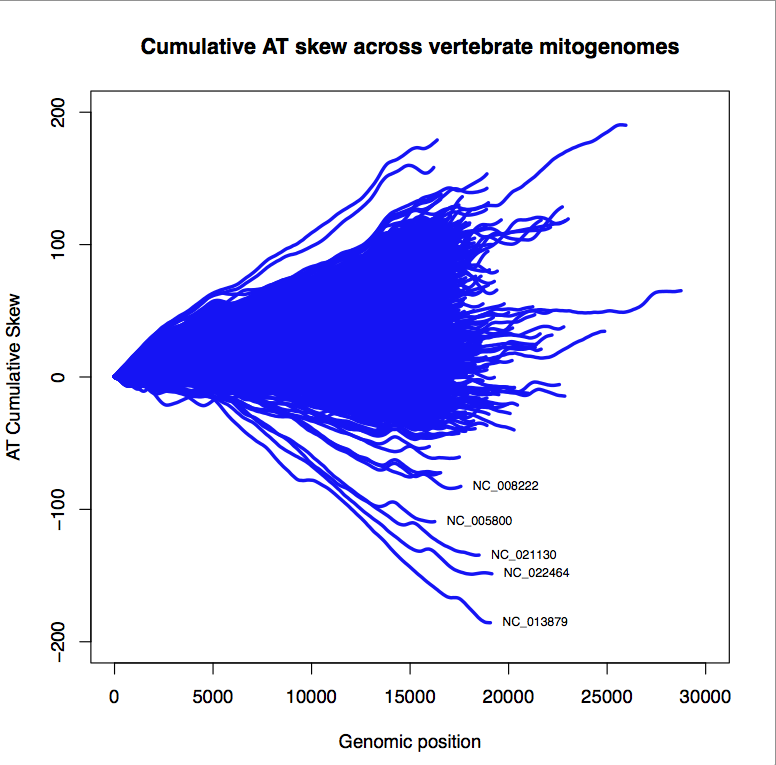

Supplement: Figure S1 — Plot of cumulative AT skew along the L-strand of 2726 vertebrate mitochondrial genomes. The values were calculated according to the formula described in Grigoriev 1998, with a window size of 500 nucleotides and a window step of 25 nucleotides. Each line corresponds to the cumulative skew of an individual mitochondrial genome. We took into account the circularity nature of the molecule in this analysis. The sequences with Control Region inversion coupled with relevant inverted compositional bias at 4-fold sites are indicated: Albula glossodonta (Albuliformes: Albulidae, NCBI code NC_005800), Bathygadus antrodes (Gadiformes: Macrouridae, NCBI code NC_008222), Tetrabrachium ocellatum (Lophiiformes: Tetrabrachiidae, NCBI code NC_013879), two Johnius species (Perciformes: Sciaenidae, J. grypotus and J. belangerii, NCBI codes NC_021130 and NC_022464, respectively). (DOCX) [file pone.0106654.s001.docx]

**Figure S2.**


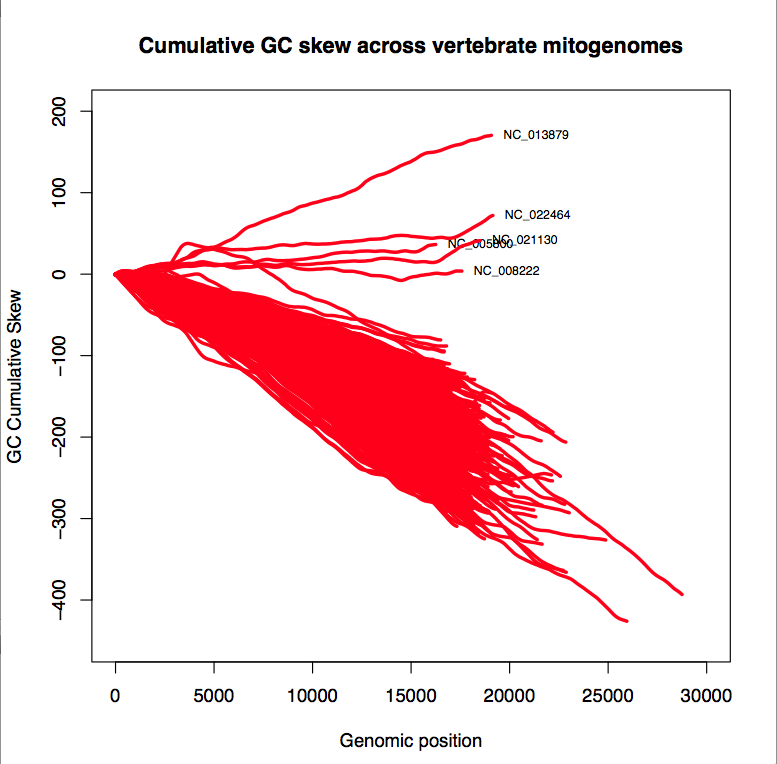

Supplement: Figure S2 — Plot of cumulative GC skew along the L-strand of 2726 vertebrate mitochondrial genomes. The values were calculated according to the formula described in Grigoriev 1998, with a window size of 500 nucleotides and a window step of 25 nucleotides. Each line corresponds to the cumulative skew of an individual mitochondrial genome. We took into account the circularity nature of the molecule in this analysis. The sequences with Control Region inversion coupled with relevant inverted compositional bias at 4-fold sites are indicated: Albula glossodonta (Albuliformes: Albulidae, NCBI code NC_005800), Bathygadus antrodes (Gadiformes: Macrouridae, NCBI code NC_008222), Tetrabrachium ocellatum (Lophiiformes: Tetrabrachiidae, NCBI code NC_013879), two Johnius species (Perciformes: Sciaenidae, J. grypotus and J. belangerii, NCBI codes NC_021130 and NC_022464, respectively). (DOCX) [file pone.0106654.s002.docx]
